# How Is Adolescent Bone Mass and Density Influenced by Early Life Body Size and Growth? The Tromsø Study: Fit Futures—A Longitudinal Cohort Study From Norway

**DOI:** 10.1002/jbm4.10049

**Published:** 2018-06-07

**Authors:** Elin Evensen, Guri Skeie, Tom Wilsgaard, Tore Christoffersen, Elaine Dennison, Anne‐Sofie Furberg, Guri Grimnes, Anne Winther, Nina Emaus

**Affiliations:** ^1^ Department of Clinical Research University Hospital of North Norway Tromsø Norway; ^2^ Department of Health and Care Sciences Faculty of Health Sciences UiT The Arctic University of Norway Tromsø Norway; ^3^ Department of Community Medicine Faculty of Health Sciences UiT The Arctic University of Norway Tromsø Norway; ^4^ Finnmark Hospital Trust Alta Norway; ^5^ MRC Lifecourse Epidemiology Unit Southampton UK; ^6^ Victoria University Wellington New Zealand; ^7^ Department of Microbiology and Infection Control University Hospital of North Norway Tromsø Norway; ^8^ Endocrinology Research Group Institute of Clinical Medicine UiT The Arctic University of Norway Tromsø Norway; ^9^ Division of Internal Medicine University Hospital of North Norway Tromsø Norway; ^10^ Division of Neurosciences Orthopedics, and Rehabilitation Services University Hospital of North Norway Tromsø Norway

**Keywords:** BIRTH WEIGHT, CHILDHOOD BMI, BONE MINERAL DENSITY

## Abstract

The effect of birth weight and childhood body mass index (BMI) on adolescents’ bone parameters is not established. The aim of this longitudinal, population‐based study was to investigate the association of birth weight, childhood BMI, and growth, with adolescent bone mass and bone density in a sample of 633 adolescents (48% girls) from The Tromsø Study: Fit Futures. This population‐based cohort study was conducted in 2010–2011 and 2012–2013 in Tromsø, Norway. Bone mineral content (BMC) and areal BMD (aBMD) were measured at total hip (TH) and total body (TB) by dual‐energy X‐ray absorptiometry (DXA) and converted to internal *Z*‐scores. Birth weight and childhood anthropometric measurements were retrospectively obtained from the Medical Birth Registry of Norway and childhood health records. Associations between birth weight, BMI, and growth were evaluated by fitting linear mixed models with repeated measures of BMC and aBMD at ages 15 to 17 and 18 to 20 years as the outcome. In crude analysis, a significant positive association (*p* < 0.05) with TB BMC was observed per 1 SD score increase in birth weight, observed in both sexes. Higher rate of length growth, conditioned on earlier size, from birth to age 2.5 years, and higher rate of weight gain from ages 6.0 to 16.5 years, conditioned on earlier size and concurrent height growth, revealed stronger associations with bone accrual at ages 15 to 20 years compared with other ages. Compared with being normal weight, overweight/obesity at age 16.5 years was associated with higher aBMD *Z*‐scores: β coefficient (95% confidence interval [CI]) of 0.78 (0.53, 1.03) and 1.08 (0.85, 1.31) in girls, 0.63 (0.42, 0.85) and 0.74 (0.54, 0.95) in boys at TH and TB, respectively. Similar associations were found for BMC. Being underweight was consistently negatively associated with bone parameters in adolescence. In conclusion, birth weight influences adolescent bone mass but less than later growth and BMI in childhood and adolescence. © 2018 The Authors. *JBMR Plus* Published by Wiley Periodicals, Inc. on behalf of the American Society for Bone and Mineral Research

## Introduction

Osteoporotic fractures constitute an important public health problem worldwide.^1^ Peak bone mass is one of several determinants of adult bone strength.^2^, ^3^ Preventive strategies have mainly focused on reducing age‐related bone loss and preventing fractures among the elderly. However, early‐life factors and optimization of peak bone mass are important factors to consider.^4^, ^5^ Maximizing peak bone mass may contribute to risk reduction of later osteoporotic fracture.^4^ A combination of genetic, hormonal, environmental, and lifestyle factors influence skeletal development,^2^, ^3^, ^6^, ^7^ and lifestyle factors may contribute to 20% to 40% of variance in adult peak bone mass.^6^, ^7^ The foundation of bone strength is laid in utero,^3^, ^8^ and subsequent growth in infancy, childhood, and adolescence is important for the acquisition of adult peak bone mass.^3^, ^8^ Several studies have shown a positive relationship between birth weight and bone mass in children^9^ and adults,^8^, ^10^ supporting the intrauterine programming hypothesis, whereas associations between birth weight and bone strength parameters in adolescence/young adulthood have varied.^9^, ^11^ Thinness and low growth rate in childhood have been associated with an increased risk of hip fracture later in life.^12^, ^13^ Previous studies on birth weight and growth during infancy might not be representative of the growth of children today^8^, ^12^ because of the rapidly increasing prevalence of childhood overweight and obesity.^14^ A recent review concluded that overweight and obese children have a significantly higher areal bone mineral density (aBMD) than normal‐weight children, possibly because of increased mechanical loading, but the long‐term impact is not clear.^15^ By contrast, other studies have reported reduced bone mass and bone area and an increased risk of fracture among overweight and obese children.^16^, ^17^ The impact of overweight and obesity on skeletal development during growth is still uncertain, and more longitudinal studies are warranted.^9^, ^15^, ^17^, ^18^, ^19^ Our study population was born between 1992 and 1994, a period with a high mean birth weight in Norway.^20^ An increasing prevalence of overweight and obesity among Norwegian children and adolescents was also observed in the last decades.^21^ The main aims of this study were therefore 1) to explore the relationship between both birth weight and childhood body mass index (BMI) and adolescent bone mass and bone density; and 2) to investigate any differences in adolescent bone mass and density related to childhood growth. We hypothesized that higher birth weight as well as high growth rate and higher childhood BMI would be positively associated with adolescent bone strength parameters, however with a possible threshold for BMI.

## Materials and Methods

### Study population

**Figure 1:**
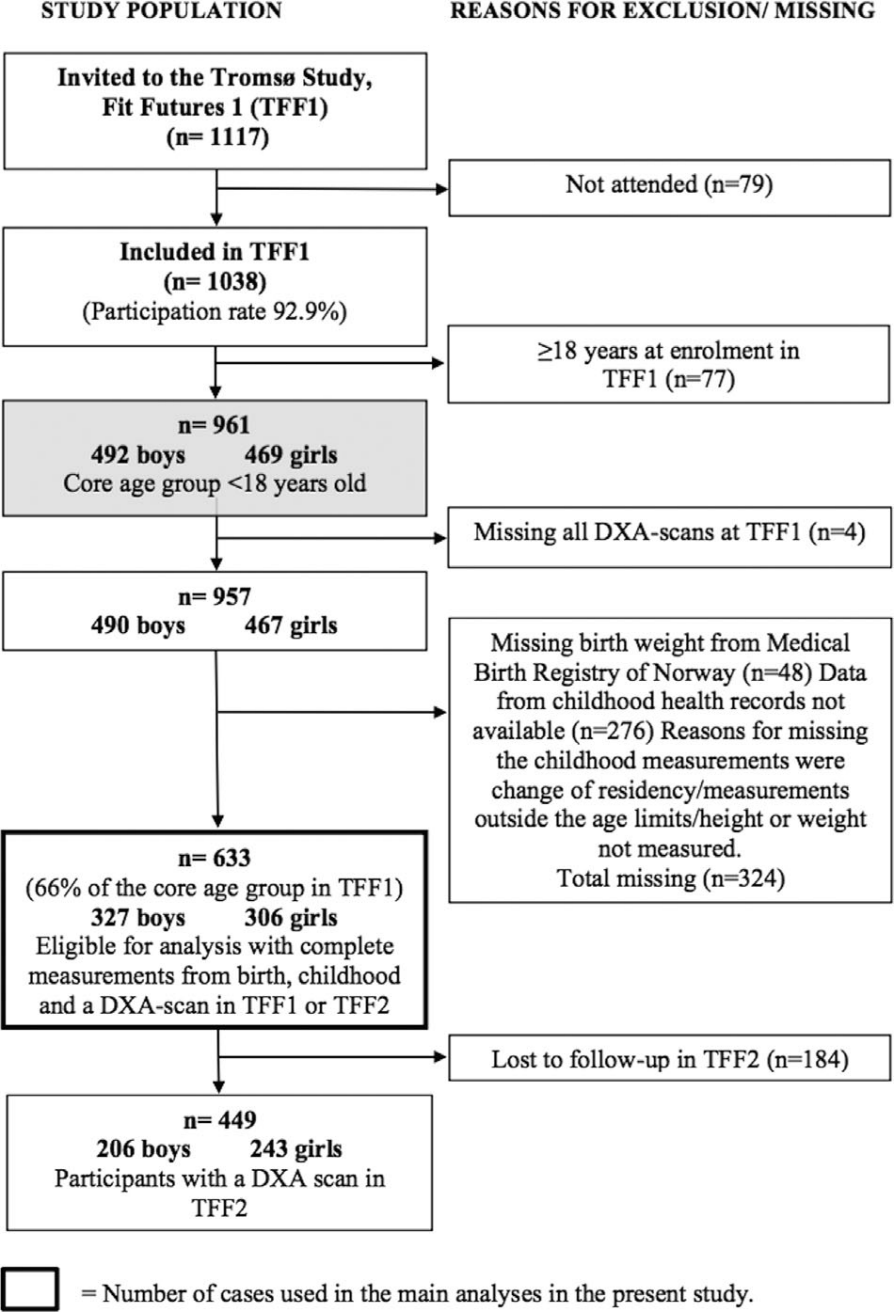
Flow chart of the study population, The Tromsø Study: Fit Futures 1 and 2.

The Tromsø Study: Fit Futures is a population‐based study with repeated health surveys among adolescents in Northern Norway. All first‐year students in Tromsø and neighboring municipalities attending upper‐secondary schools in 2010–2011 (*n* = 1117) were invited to Fit Futures 1 (TFF1); 1038 students (92.9%) attended. Among these students, 961 were in the core age group of 15 to 17 years (born 1992–1994). A follow‐up study, Fit Futures 2 (TFF2), was conducted 2 years later (2012–2013) and reinvited all participants from TFF1. Detailed information on TFF1 and TFF2 has been presented earlier.^22^, ^23^ Data from the cohort were supplemented with retrospectively collected anthropometric data from birth and childhood. A sample of 633 participants (48% girls), with measurements from birth, childhood, and one or two dual‐energy X‐ray absorptiometry (DXA) measurements from ages 15 to 17 and 18 to 20 years was eligible for the analysis in the present study (a flowchart is shown in Fig. 1). This constitutes 66% of the 961 students in the core age group in TFF1. The Regional Committee for Medical and Health Research Ethics, North Norway (REK nord) approved TFF1, TFF2, and the present study (reference number: 2014/1397/REK nord). All students and parents/guardians of students age <16 years gave written informed consent.

### Bone mass and bone density at ages 15 to 17 and 18 to 20 years

Bone mass and bone density in this study were measured as total hip (TH) and total body (TB) bone mineral content (BMC; g) and aBMD (g/cm^2^) by DXA (GE Lunar Prodigy, Lunar Corporation, Madison, WI, USA) and analyzed with Encore pediatric software version 13.4. In vivo, the densitometer coefficient of variation for TH was estimated at 1.17%.^24^ Repeated measurements were performed in TFF1 and TFF2 with the same DXA instrument, and all measurements from the same wave were analyzed by a single investigator. The left‐side values were used as an outcome measure in the analyses. In case of missing data or error, the right‐side values from both TFF1 and TFF2 were used. We converted the bone measures to sex‐ and age‐standardized internal *Z*‐scores based on the distribution of the study sample.

Height and weight in TFF1 and TFF2 were measured to the nearest 0.1 cm and 0.1 kg, respectively, on an automatic electronic stadiometer/scale (Jenix DS 102, Dong Sahn Jenix, Seoul, Korea). Participants wore light clothing and no footwear. Trained study nurses at the Clinical Research Unit, University Hospital of North Norway, performed DXA and all anthropometric measurements, following standardized procedures.

### Measures from birth and childhood

Information on birth weight (g), length (cm), and gestational age (GA; weeks) were obtained through linkage to the Medical Birth Registry of Norway (MBRN) using participants’ unique personal identification number. GA was determined by ultrasound examination or last menstrual period if ultrasound was missing. Anthropometric measurements are part of regular health examinations by public health nurses in accordance with national preventive health program guidelines. Therefore, we were able to retrospectively collect data on height (cm), weight (kg), age (years, months), and date of measurements at two time points (target ages; 2 and 6 years of age) from childhood health records. The exact age of the participants at the time measurements were taken varied slightly (median age 2.5 years, range 1.9 to 4.5 years and median age 6.0 years, range 5.0 to 7.6 years).

### Estimating length/height and weight growth trajectories

Because not all participants were measured at exact same age, we used linear spline multilevel model^25^ to estimate each participant's height (cm) and weight (kg) at the ages 2.5, 6.0, and 16.5 years. The model, also referred to as “the broken stick model,”^25^ use data from individuals and from the whole study sample to estimate person‐specific birth weight, length/height, and weight with knots at the target ages 2.5, 6.0, and 16.5 years, and length/height and weight growth trajectories between consecutive ages. In our study, each participant had only one collected height/weight measurement around the target ages and knot points were therefore placed at the median ages. Individual‐level random effects for intercept and slopes are estimated as each person's deviation from the average trajectory.^25^ Sex and an interaction term with sex and splines were included in the model to account for sex differences in growth trajectories over time. Five percent of participants were missing length at birth, and missing values were predicted with this model. In a two‐step process, model estimates were used for further calculation of exposure variables that were used in our analysis of the outcome measures. Models were fitted using the mixed command in Stata.^25^ Length/height and weight growth rate were calculated as change in cm/kg per year between two consecutive target ages, eg, predicted height at 16.5 years of age minus predicted height at 6.0 years of age divided by 10.5 years.

### Exposure variables

Sex‐specific birth weight and length standard deviation scores (SDS) were calculated according to GA, using the British 1990 growth reference.^26^

Based on BMI (predicted weight [kg]/predicted height [m^2^]) at 2.5, 6.0, and 16.5 years, participants were categorized into the following BMI categories: underweight (corresponding to adult BMI <18.5 kg/m^2^), normal weight (adult BMI ≥18.5 to <25 kg/m^2^), and overweight/obesity (adult BMI ≥25 kg/m^2^). Because of a relatively small proportion of obesity at these ages, we merged the overweight and obese category. Sex‐specific BMI reference values at the target ages were used according to the International Obesity Taskforce age‐ and sex‐specific cut‐off values for children ages 2 to 18 years.^27^

### Covariates from questionnaires in TFF1

Information regarding ethnicity, pubertal maturation, and physical activity was taken from self‐administered questionnaires completed during TFF1. Girls were categorized into three stages of pubertal maturation: early (<12.5 years), intermediate (12.5 to 13.9 years), and late (≥14.0 years), based on age at menarche. Pubertal maturation in boys was classified as barely started, underway, and completed based on the pubertal development scale (PDS). The boys rated four secondary sexual characteristics on a scale ranging from 1 (not yet started) to 4 (complete) and the PDS score was calculated as a total mean score of the four items.^22^, ^28^ Physical activity frequency was measured through the validated WHO Health Behaviour in Schoolchildren (HBSC) questionnaire,^29^ which included the question: “If you are actively doing sports or physical activity outside school, how many days a week are you active?” Answers were given in six predefined categories; “never” (1), “less than once a week” (2), “1 day a week” (3), “2 to 3 days a week” (4), “4 to 6 days a week” (5), and “almost every day” (6). The answers were recoded into three categories of physical activity: “low” (1–2), “moderate” (3–4), and “high” (5–6).

### Statistical analyses

Characteristics of the study population are presented as means and standard deviations (SD) or numbers and percentages for girls and boys separately. ANOVA with the Bonferroni correction for multiple comparisons was used to assess differences in mean height according to BMI category. The main outcomes in the present study were TH and TB standardized BMC and aBMD scores (*Z*‐scores) at ages 15 to 17 and 18 to 20 years. In a two‐step process, we used linear spline multilevel model^25^ to predict each participant's height and weight at exact ages 2.5, 6.0, and 16.5 years. In the second step, linear mixed models with a random intercept on the subject level were used to evaluate the relationship between birth weight SDS, BMI category at 2.5, 6.0, and 16.5 years of age, height and weight growth rate, and repeated BMC and aBMD *Z*‐scores as continuous outcomes. Associations of birth weight SDS, or BMI category, with BMC/aBMD *Z*‐scores as outcomes were assessed using the following models: 1) unadjusted; 2) birth weight SDS adjusted for GA and birth length; 3) BMI category adjusted for height at 2.5 and 6.0 years of age, respectively; 4) BMI category at 16.5 years of age adjusted for height at same age, pubertal maturation, and physical activity, as potential confounding factors. In accordance with others,^30^, ^31^ associations of length/height and weight growth rate with BMC/aBMD outcomes were assessed using the following models: 1) models of length/height growth were adjusted for birth weight, length/height at the beginning of each period and preceding length/height growth rate; 2) models of weight gain were adjusted for birth weight, length/height, and weight at the beginning of each period and length/height growth rate over the same time span. The models from birth to age 2.5 years were additionally adjusted for GA. The models from ages 6.0 to 16.5 years were additionally adjusted for pubertal maturation.

Because bone growth, magnitude, and tempo of bone acquisition differ between girls and boys, especially in adolescence,^3^, ^4^ all analyses were stratified by sex. Cross‐product terms with sex and exposure variables were included in the models to formally test for potential sex interactions. No statistically significant sex difference was observed. Maternal age at birth and age at outcome measurement was included as potential confounders. However, they did not affect the estimated coefficients and were not included in the final models. Normality and linearity of exposures and outcomes and residuals were checked by visual inspections of histograms and plots. There were signs of nonlinearity between BMI at age 16.5 years and bone measurements (Supplemental Fig. S1). A formal test with a quadratic term of BMI at age 16.5 years in the final model confirmed a nonlinear relationship with TB BMC and aBMD. BMI in categories were therefore used as the exposure variable. For ease of comparison, BMI categories were used at all ages. No assumptions were considered violated for the final models.

We experienced additional missing data in covariates, and to avoid bias, we performed multiple imputations (20 imputations) of missing values using chained equations.^32^ The percentage of missing covariates in the study sample were 20% in total; 9% for GA, 1% for physical activity, 11% for PDS in boys, and <1% for menarche age in girls. The values were imputed based on observed data from all 633 participants. The imputation model included all variables from the final adjusted models, BMC and aBMD outcome variables, in addition to auxiliary data from the MBRN. Sensitivity analyses were performed in the data set with no imputations (*n* = 633 only observed values) and in a complete‐case data set (*n* = 367). The results were similar and the results of mixed model analyses presented here are from the imputed data sets. In a dropout analysis, we explored differences between participants with and without missing data by *t* test for continuous variables and by the chi‐square test for categorical variables. Multiple imputations, linear spline multilevel models, and statistical analyses were all carried out using Stata/MP 14.2 for Mac (StataCorp., College Station, TX, USA). The level of statistical significance was set to two‐sided *p* values of <0.05.

## Results

### Characteristics of the study population

In the present study, we used data from 327 boys and 306 girls with measurements from birth and childhood, and DXA measurements from 15 to 17 years of age. Seventy‐one percent of our study sample (206 boys and 243 girls) also had DXA measures from TFF2 (Fig. 1). The dropout analysis showed no significant difference in mean values of the exposure variables or outcome variables at TFF1, between those participating at TTF2, and those lost to follow‐up. Nor were significant differences observed in mean BMC/aBMD variables for participants missing childhood exposure variables. However, significantly more boys than girls were lost to follow‐up (data not shown). The majority (98%) of our study sample was of white ethnicity. Characteristics for the study population from birth to 18 to 20 years of age, with predicted length/height, weight, and BMI measures from birth to 16.5 years of age, are shown in Table 1. Mean birth weight was 3480 g among girls and 3575 g among boys. The prevalence of overweight/obesity was 11.4% and 17.6% in girls, and 7.9% and 10.4% in boys at 2.5 and 6.0 years of age, respectively. At age 16.5 years, 22% of girls and boys were overweight/obese. Differences between observed and predicted length/height and weight values and calculated BMI at target ages were small and are shown in Supplemental Table S1. Characteristics for variables with missing and imputed values are shown in Supplemental Table S2. Results of ANOVA showed no significant difference in height at 15 to 17 or 18 to 20 years of age between the three BMI categories at 2.5, 6.0, or 16.5 years of age, neither in girls nor boys (data not shown).

**Table 1:** Sex‐Specific Characteristics of the Study Population at Birth and Four Ages Up to 18 to 20 Years (The Tromsø Study: Fit Futures)

	Girls			Boys		
Characteristics^a^	*n*	Mean/%	SD	*n*	Mean/%	SD
Birth						
Birth weight (g)	306	3480.6	554.3	327	3575.2	566.7
Birth length (cm)	306	49.5	1.6	327	50.1	1.8
Gestational age (weeks)	272	39.8	1.8	303	39.5	2.1
Birth weight SDS^b^	306	0.24	1.03	327	0.26	1.04
Birth length SDS^b^	306	−0.37	0.81	327	−0.33	0.82
2.5 years of age						
Weight (kg)	306	13.6	1.6	327	14.2	1.6
Height (cm)	306	91.6	3.2	327	93.1	3.3
Body mass index (kg/m^2^)	306	16.11	1.37	327	16.32	1.30
BMI category^c^	306			327		
Underweight	49	16.0%		47	14.4%	
Normal weight	222	72.6%		254	77.7%	
Overweight/obese	35	11.4%		26	7.9%	
6.0 years of age						
Weight (kg)	306	21.6	3.8	327	21.6	3.3
Height (cm)	306	117.1	4.2	327	118.0	4.5
Body mass index (kg/m^2^)	306	15.69	2.20	327	15.48	1.79
BMI category^c^	306			327		
Underweight	48	15.7%		51	15.6%	
Normal weight	204	66.7%		242	74.0%	
Overweight/obese	54	17.6%		34	10.4%	
16.5 years of age						
Weight (kg)	306	61.9	12.3	327	69.9	14.6
Height (cm)	306	166.1	6.0	327	177.7	6.6
Body mass index (kg/m^2^)	306	22.44	4.32	327	22.07	4.20
BMI category^c^	306			327		
Underweight	20	6.5%		31	9.5%	
Normal weight	218	77.8%		224	68.5%	
Overweight/obese	68	22.2%		72	22.0%	
BMC total hip (g)	305	32.1	4.7	327	39.8	6.6
BMC total body (g)	306	2545.3	392.6	327	2936.7	462.4
aBMD total hip (g/cm^2^)	305	1.06	0.12	327	1.11	0.15
aBMD total body (g/cm^2^)	306	1.14	0.08	327	1.18	0.09
Pubertal maturation,^d^ girls	303			—	—	
Early (<12.5 years)	85	28.1%		—	—	
Intermediate (12.5–13.9 years)	152	50.2%		—	—	
Late (≥14.0 years)	66	21.8%		—	—	
Pubertal maturation,^e^ boys	—	—		259		
Barely started (PDS 2.0–2.9)	—	—		46	17.8%	
Underway (PDS 3.0–3.9)	—	—		196	75.7%	
Completed (PDS 4.0)	—	—		17	6.6%	
Physical activity–frequency	303			321		
Low	103	34.0%		118	36.8%	
Moderate	133	43.9%		117	36.5%	
High	67	22.1%		86	26.8%	
18–20 years of age						
Age (years)	259	18.6	0.4	211	18.7	0.4
Weight (kg)	246	63.7	12.3	206	74.4	14.4
Height (cm)	246	166.3	6.2	206	179.5	6.7
Body mass index (kg/m^2^)	246	23.07	4.57	206	23.07	4.08
BMC total hip (g)	242	32.4	4.8	206	41.4	7.2
BMC total body (g)	243	2612.1	377.3	206	3190.7	473.0
aBMD Total hip (g/cm^2^)	242	1.07	0.13	206	1.15	0.16
aBMD Total body (g/cm^2^)	243	1.15	0.07	206	1.23	0.09

SD = standard deviation; SDS = standard deviation scores; BMI = body mass index; BMC = bone mineral content; aBMD = areal bone mineral density; PDS = pubertal development scale.

^a^ Birth weight, birth length, weight, and height are predicted mean values (SD) at target ages (2.5, 6.0, and 16.5 years), using linear spline multilevel model. Birth weight and length SDS, BMI, and BMI category at the target ages are based on these predicted values. The rest of the characteristics are observed values measured between 15 to 17 and 18 to 20 years of age.

^b^ SDS according to British 1990 growth charts, version UK.^26^

^c^ BMI categories according to International Obesity Taskforce age‐ and sex‐specific cut‐off values for children 2–18 years of age; underweight (adult BMI <18.5 kg/m^2^), normal weight (adult BMI ≥18.5–<25 kg/m^2^), overweight obesity (adult BMI ≥25 kg/m^2^).^27^

^d^ Pubertal maturation is based on age of menarche in girls.

^e^ PDS in boys; total score of four items of secondary sexual characteristics on a scale from 1 to 4 (sum of total score divided by 4 (none had a score <2.0 in total score).^28^

### Birth weight and bone measures

In crude analyses, 1 SD score higher birth weight was significantly associated with 0.31 (95% confidence interval [CI] 0.20 to 0.41, *p* < 0.001) higher TB BMC *Z*‐scores at 15 to 20 years of age in girls, and 0.13 (95% CI 0.02 to 0.23, *p* = 0.017) higher TB BMC *Z*‐scores in boys (Tables 2 and 3). In girls, significant associations were also found with TH BMC and TB aBMD *Z*‐scores (Table 2). After additional adjustment for length at birth, the association attenuated, except for TB BMC in girls. However, no statistically significant interaction was found between sex and birth weight.

**Table 2:** Associations of Birth Weight and BMI Category^a^ at 2.5, 6.0, and 16.5 Years of Age With Bone Mineral Content and Bone Mineral Density at 15 to 20 Years of Age in Girls (The Tromsø Study: Fit Futures)

	Total hip		Total body		Total hip		Total body	
	Crude models				Adjusted models^b^			
Girls (*n* = 306)	β (95% CI)	*p* Value	β (95% CI)	*p* Value	β (95% CI)	*p* Value	β (95% CI)	*p* Value
Bone mineral content								
Birth								
Birth weight SDS^c^	0.19 (0.08, 0.29)	0.001	0.31 (0.20, 0.41)	<0.001	0.09 (−0.06, 0.24)	0.220	0.21 (0.07, 0.35)	0.004
2.5 years of age								
Underweight	−0.27 (−0.58, 0.03)	0.079	−0.49 (−0.79, −0.20)	0.001	−0.26 (−0.55, 0.03)	0.080	−0.47 (−0.74, −0.21)	<0.001
Normal weight	Ref.		Ref.		Ref.		Ref.	
Overweight/obese	0.29 (−0.06, 0.64)	0.104	0.50 (0.16, 0.84)	0.004	0.20 (−0.14, 0.54)	0.243	0.37 (0.06, 0.68)	0.018
6.0 years of age								
Underweight	−0.28 (−0.58, 0.02)	0.064	−0.49 (−0.77, −0.20)	0.001	−0.23 (−0.52, 0.05)	0.110	−0.41 (−0.66, −0.17)	0.001
Normal weight	Ref.		Ref.		Ref.		Ref.	
Overweight/obese	0.64 (0.35, 0.93)	<0.001	0.89 (0.62, 1.16)	<0.001	0.51 (0.23, 0.78)	<0.001	0.70 (0.46, 0.93)	<0.001
16.5 years of age								
Underweight	−0.56 (−0.98, −0.15)	0.008	−0.79 (−1.16, −0.42)	<0.001	−0.47 (−0.85, −0.09)	0.016	−0.72 (−1.03, −0.41)	<0.001
Normal weight	Ref.		Ref.		Ref.		Ref.	
Overweight/obese	0.91 (0.66, 1.15)	<0.001	1.22 (1.00, 1.44)	<0.001	1.05 (0.83, 1.28)	<0.001	1.34 (1.16, 1.53)	<0.001
Areal bone mineral density								
Birth								
Birth weight SDS^c^	0.07 (−0.04, 0.18)	0.236	0.14 (0.03, 0.25)	0.014	0.07 (−0.08, 0.22)	0.353	0.10 (−0.05, 0.25)	0.181
2.5 years of age								
Underweight	−0.18 (−0.48, 0.13)	0.261	−0.39 (−0.69, −0.08)	0.012	−0.17 (−0.48, 0.13)	0.264	−0.38 (−0.67, −0.08)	0.013
Normal weight	Ref.		Ref.		Ref.		Ref.	
Overweight/obese	0.12 (−0.23, 0.47)	0.501	0.35 (−0.00, 0.69)	0.051	0.11 (−0.24, 0.47)	0.529	0.29 (−0.05, 0.63)	0.098
6.0 years of age								
Underweight	−0.34 (−0.65, −0.04)	0.027	−0.51 (−0.80, −0.22)	<0.001	−0.34 (−0.64, −0.03)	0.030	−0.48 (−0.76, −0.20)	0.001
Normal weight	Ref.		Ref.		Ref.		Ref.	
Overweight/obese	0.47 (0.18, 0.76)	0.001	0.82 (0.55, 1.10)	<0.001	0.45 (0.16, 0.75)	0.002	0.74 (0.46, 1.01)	<0.001
16.5 years of age								
Underweight	−0.47 (−0.90, −0.04)	0.031	−0.80 (−1.20, −0.41)	<0.001	−0.28 (−0.71, 0.15)	0.203	−0.56 (−0.94, −0.17)	0.005
Normal weight	Ref.		Ref.		Ref.		Ref.	
Overweight/obese	0.69 (0.44, 0.95)	<0.001	0.99 (0.76, 1.23)	<0.001	0.78 (0.53, 1.03)	<0.001	1.08 (0.85, 1.31)	<0.001

BMI = body mass index; CI = confidence interval; SDS = standard deviation scores.

Values are based on linear mixed models and reflect standardized β coefficients for bone mineral content and areal bone mineral density, 95% CI, and corresponding *p* value. Models at birth and at 16.5 years of age are based on data with multiple imputation of missing covariates.

^a^ BMI category according to International Obesity Taskforce age‐ and sex‐specific cut‐off values for children 2 to 18 years of age; underweight (adult BMI <18.5 kg/m^2^), normal weight (adult BMI ≥18.5–<25 kg/m^2^), overweight/obesity (adult BMI ≥25 kg/m^2^).^27^

^b^ Birth weight standard deviation score are additionally adjusted for gestational age and birth length. Childhood models are adjusted for height at 2.5 and 6.0 years of age, respectively. Model at 16.5 years of age are adjusted for height at same age, pubertal maturation and physical activity–frequency measured in Fit Futures 1.

^c^ Sex‐specific birth weight standard deviation scores are adjusted for gestational age and calculated according to British 1990 growth charts, version UK.^26^

**Table 3:** Associations of Birth Weight and BMI Category^a^ at 2.5, 6.0, and 16.5 Years of Age With Bone Mineral Content and Bone Mineral Density at 15 to 20 Years of Age in Boys (The Tromsø Study: Fit Futures)

	Total hip		Total body		Total hip		Total body	
	Crude models				Adjusted models^b^			
Boys (*n* = 327)	β (95% CI)	*p* Value	β (95% CI)	*p* Value	β (95% CI)	*p* Value	β (95% CI)	*p* Value
Bone mineral content								
Birth								
Birth weight SDS^c^	0.03 (−0.07, 0.13)	0.579	0.13 (0.02, 0.23)	0.017	−0.12 (−0.27, 0.03)	0.126	−0.08 (−0.23, 0.07)	0.291
2.5 years of age								
Underweight	−0.06 (−0.36, 0.24)	0.696	−0.16 (−0.47, 0.14)	0.289	−0.06 (−0.34, 0.22)	0.688	−0.16 (−0.42, 0.10)	0.222
Normal weight	Ref.		Ref.		Ref.		Ref.	
Overweight/obese	0.21 (−0.18, 0.61)	0.291	0.45 (0.06, 0.84)	0.025	0.04 (−0.33, 0.41)	0.826	0.21 (−0.13, 0.55)	0.233
6.0 years of age								
Underweight	−0.20 (−0.49, 0.09)	0.184	−0.31 (−0.60, −0.02)	0.036	−0.12 (−0.38, 0.15)	0.388	−0.20 (−0.43, 0.04)	0.099
Normal weight	Ref.		Ref.		Ref.		Ref.	
Overweight/obese	0.42 (0.08, 0.77)	0.017	0.68 (0.34, 1.02)	<0.001	0.33 (0.01, 0.64)	0.043	0.55 (0.27, 0.82)	<0.001
16.5 years of age								
Underweight	−0.73 (−1.07, −0.39)	<0.001	−0.90 (−1.22, −0.59)	<0.001	−0.54 (−0.82, −0.27)	<0.001	−0.80 (−1.02, −0.58)	<0.001
Normal weight	Ref.		Ref.		Ref.		Ref.	
Overweight/obese	0.67 (0.43, 0.91)	<0.001	0.97 (0.74, 1.19)	<0.001	0.67 (0.48, 0.86)	<0.001	0.94 (0.78, 1.10)	<0.001
Areal bone mineral density								
Birth								
Birth weight SDS^c^	−0.02 (−0.12, 0.09)	0.732	0.02 (−0.08, 0.13)	0.651	−0.13 (−0.28, 0.02)	0.096	−0.10 (−0.25, 0.05)	0.191
2.5 years of age								
Underweight	−0.05 (−0.35, 0.25)	0.747	−0.15 (−0.46, 0.15)	0.319	−0.05 (−0.34, 0.25)	0.748	−0.15 (−0.44, 0.14)	0.300
Normal weight	Ref.		Ref.		Ref.		Ref.	
Overweight/obese	0.09 (−0.31, 0.48)	0.668	0.20 (−0.19, 0.60)	0.307	−0.02 (−0.40, 0.37)	0.924	0.06 (−0.32, 0.43)	0.766
6.0 years of age								
Underweight	−0.15 (−0.44, 0.14)	0.302	−0.22 (−0.50, 0.07)	0.138	−0.11 (−0.39, 0.18)	0.459	−0.15 (−0.42, −0.12)	0.274
Normal weight	Ref.		Ref.		Ref.		Ref.	
Overweight/obese	0.31 (−0.03, 0.66)	0.075	0.64 (0.30, 0.99)	<0.001	0.26 (−0.08, 0.60)	0.130	0.57 (0.24, 0.89)	0.001
16.5 years of age								
Underweight	−0.60 (−0.94, −0.25)	0.001	−0.90 (−1.23, −0.58)	<0.001	−0.38 (−0.69, −0.08)	0.013	−0.74 (−1.03, −0.45)	<0.001
Normal weight	Ref.		Ref.		Ref.		Ref.	
Overweight/obese	0.61 (0.37, 0.85)	<0.001	0.74 (0.50, 0.97)	<0.001	0.63 (0.42, 0.85)	<0.001	0.74 (0.54, 0.95)	<0.001

BMI = body mass index; CI = confidence interval; SDS = standard deviation scores.

Values are based on linear mixed models and reflect standardized β coefficients for bone mineral content and areal bone mineral density, 95% CI, and corresponding *p* value. Models at birth and at 16.5 years of age are based on data with multiple imputation of missing covariates.

^a^ BMI category according to International Obesity Taskforce age‐ and sex‐specific cut‐off values for children 2 to 18 years of age; underweight (adult BMI <18.5 kg/m^2^), normal weight (adult BMI ≥18.5 to <25 kg/m^2^), overweight/obesity (adult BMI ≥25 kg/m^2^).^27^

^b^ Birth weight standard deviation score are additionally adjusted for gestational age and birth length. Childhood models are adjusted for height at 2.5 and 6.0 years of age, respectively. Model at 16.5 years of age are adjusted for height at same age, pubertal maturation, and physical activity–frequency measured in Fit Futures 1.

^c^ Sex‐specific birth weight standard deviation score are adjusted for gestational age and calculated according to British 1990 growth charts, version UK.^26^

### BMI category and bone measures

We found a pattern of increasing TH and TB BMC/aBMD *Z*‐scores with higher BMI category. Stronger associations were found with advancing age (Tables 2 and 3). In both sexes, in crude analyses and analyses adjusted for height, overweight/obesity at 6.0 years of age was associated with higher TH and TB BMC at 15 to 20 years of age compared with those of normal weight. Significant associations were also found with TB aBMD in both sexes and with TH aBMD only in girls. In both sexes, crude analysis revealed a significant association between overweight/obesity at 2.5 years of age and higher TB BMC values at age 15 to 20 years. In analyses adjusted for current height, pubertal maturation, and physical activity, overweight/obesity at 16.5 years of age was associated with >1.0 *Z*‐score higher TH and TB BMC among girls. Positive associations were also found for aBMD: TH aBMD *Z*‐scores 0.78 (95% CI 0.53 to 1.03) and TB aBMD 1.08 (95% CI 0.85 to 1.31) in girls. Significant positive associations with BMC/aBMD *Z*‐scores were also found among overweight/obese boys at age 16.5 years; TH aBMD 0.63 (95% CI 0.42 to 0.85), TB aBMD 0.74 (95% CI 0.54 to 0.95) (Tables 2 and 3). The effect of BMI leveled off at BMI >30 kg/m^2^ (Supplemental Figs. S1 and S2). A formal test with a quadratic term for BMI at age 16.5 years was significant for TB BMC and aBMD for both girls and boys. In both sexes, being underweight at 2.5, 6.0, or 16.5 years of age was associated with negative BMC and aBMD *Z*‐scores at 15 to 20 years of age compared with those of normal weight. The strongest negative associations were found at 16.5 years of age (Tables 2 and 3).

### Length/height and weight growth trajectories and bone measures

In analyses of individually modeled length/height growth trajectories, a 1‐SD higher length/height growth rate in early childhood (from birth to 2.5 years of age and from 2.5 to 6.0 years of age) showed positive associations with both TH and TB BMC and aBMD *Z*‐scores at 15 to 20 years of age (Table 4). Length growth rate from birth to 2.5 years of age, conditioned on earlier size, showed the strongest associations with TH and TB BMC *Z*‐scores in both sexes. A 1‐SD higher height growth rate from 6.0 to 16.5 years of age, conditioned on earlier size and growth and pubertal maturation, displayed weaker, or negative, nonsignificant associations with BMC/aBMD at 15 to 20 years of age in both girls and boys (Table 4).

**Table 4:** Associations of Length/Height and Weight Growth Rate Between Birth 2.5, 6.0, and 16.5 Years of Age With Bone Mineral Content and Bone Mineral Density at 15 to 20 Years of Age in Girls and Boys (The Tromsø Study: Fit Futures)

		Bone mineral content				Areal bone mineral density			
		Total hip		Total body		Total hip		Total body	
Per SD increase in height or weight growth rate	Mean increase/yr	β (95% CI)	*p* Value	β (95% CI)	*p* Value	β (95% CI)	*p* Value	β (95% CI)	*p* Value
Girls (*n* = 306)									
Birth to 2.5 years of age									
Length (1.1 cm/yr)^a^	16.9	0.24 (0.12, 0.35)	<0.001	0.37 (0.26, 0.47)	<0.001	0.01 (−0.11, 0.13)	0.882	0.18 (0.06, 0.30)	0.003
Weight (0.6 kg/yr)^b^	4.0	0.20 (0.06, 0.34)	0.006	0.33 (0.20, 0.45)	<0.001	0.13 (−0.02, 0.28)	0.080	0.32 (0.17, 0.46)	<0.001
2.5 to 6.0 years of age									
Height (0.6 cm/yr)^c^	7.3	0.21 (0.10, 0.32)	<0.001	0.30 (0.20, 0.40)	<0.001	0.12 (0.00, 0.24)	0.050	0.23 (0.12, 0.34)	<0.001
Weight (0.8 kg/yr)^d^	2.3	0.21 (0.10, 0.32)	<0.001	0.29 (0.20, 0.38)	<0.001	0.24 (0.12, 0.36)	<0.001	0.35 (0.24, 0.46)	<0.001
6.0 to 16.5 years of age									
Height (0.6 cm/yr)^c,^ ^e^	4.7	0.14 (−0.05, 0.33)	0.143	0.06 (−0.11, 0.23)	0.470	−0.10 (−0.30, 0.10)	0.327	−0.19 (−0.38, 0.00)	0.053
Weight (1.2 kg/yr)^d,^ ^e^	3.8	0.46 (0.33, 0.59)	<0.001	0.57 (0.48, 0.67)	<0.001	0.35 (0.21, 0.50)	<0.001	0.42 (0.29, 0.54)	<0.001
Boys (n = 327)									
Birth to 2.5 years of age									
Length (1.1 cm/yr)^a^	17.2	0.33 (0.22, 0.43)	<0.001	0.47 (0.37, 0.56)	<0.001	0.20 (0.09, 0.30)	<0.001	0.32 (0.21, 0.42)	<0.001
Weight (0.6 kg/yr)^b^	4.2	0.11 (−0.02, 0.24)	0.098	0.17 (0.06, 0.29)	0.004	0.09 (−0.05, 0.22)	0.202	0.16 (0.02, 0.29)	0.021
2.5 to 6.0 years of age^e^									
Height (0.6 cm/yr)^c^	7.1	0.22 (0.12, 0.31)	<0.001	0.28 (0.19, 0.37)	<0.001	0.11 (0.01, 0.21)	0.039	0.17 (0.07, 0.27)	0.001
Weight (0.8 kg/yr)^d^	2.1	0.16 (0.04, 0.28)	0.011	0.24 (0.13, 0.34)	<0.001	0.14 (0.01, 0.27)	0.030	0.29 (0.17, 0.42)	<0.001
6.0 to 16.5 years of age									
Height (0.6 cm/yr)^c,e^	5.7	0.20 (0.02, 0.38)	0.027	0.23 (0.08, 0.39)	0.004	−0.01 (−0.20, 0.18)	0.912	−0.02 (−0.20, 0.17)	0.843
Weight (1.2 kg/yr)^d,e^	4.6	0.30 (0.20, 0.40)	<0.001	0.41 (0.33, 0.48)	<0.001	0.27 (0.17, 0.38)	<0.001	0.33 (0.24, 0.43)	<0.001

SD = standard deviation; CI = confidence interval.

Values are based on multiple linear mixed models and reflect standardized β coefficients for bone mineral content and areal bone mineral density, 95% CI, and corresponding *p* value. Models at birth and at 16.5 years of age are based on data with multiple imputation of missing covariates.

^a^ Length growth from 0 to 2.5 years of age are adjusted for birth weight, birth length, and gestational age.

^b^ Weight growth from 0 to 2.5 years of age are adjusted for birth weight, birth length, gestational age, and length growth over the same time span.

^c^ Height growth models are adjusted for birth weight, height at start of the growth period, and preceding height growth.

^d^ Weight growth models are adjusted for birth weight, height and weight at start of the growth period, and length growth over the same time span.

^e^ Models of growth from 6.0 to 16.5 years of age are additionally adjusted for pubertal maturation.

Individually modeled rate of weight gain, conditioned on earlier size and growth and concurrent height growth, was positively associated with bone parameters at 15 to 20 years of age. Estimated coefficients increased with age for both TH and TB and BMC and aBMD. The strongest associations between 1‐SD higher rate of weight gain and bone parameters, conditioned on earlier size and growth and concurrent height growth, were found between 6.0 and 16.5 years of age (Table 4).

### Sensitivity analysis

Sensitivity analysis excluding children born preterm (4.4% born before GA week 37) or twins (3.8%) did not change the results or revealed patterns. Only minor changes in estimated coefficients were found in all analyses. Twins or preterm‐born participants were therefore not excluded from the analyses. Sensitivity analyses run on a data set with no imputations and in a complete‐case data set produced results similar to those presented.

## Discussion

In this longitudinal population‐based study of adolescents, we have explored associations between birth weight, childhood BMI, and growth rate with BMC and aBMD at 15 to 20 years of age. A significantly positive association was found between birth weight and TB BMC at 15 to 20 years of age, both in girls and boys. We observed significant associations between higher childhood BMI and greater adolescent bone mass. In both sexes, overweight/obesity at 6.0 or 16.5 years of age revealed from 0.5 to 1.1 higher *Z*‐scores for TH BMC and aBMD at 15 to 20 years of age compared with those of normal weight. The corresponding associations with TB were somewhat stronger. Being underweight during childhood and adolescence was consistently negatively associated with bone parameters at 15 to 20 years of age. In early childhood, up to 6.0 years of age, higher rates of length/height growth and weight gain were positively associated with bone mass accrual at 15 to 20 years of age. In this period, a high rate of length/height growth was more strongly associated with adolescent bone mass accrual than a high rate of weight gain. In contrast, a high rate of weight gain, but not height growth, from 6.0 to 16.5 years of age showed strong positive associations with both bone mass and density.

Childhood and adolescence represent a critical window of opportunity for lifestyle interventions to maximize bone mass.^4^ Information on how BMI and growth in childhood influence later peak bone mass is important, especially in times of increasing childhood overweight and obesity. Our study brings updated results on this relationship from a country and a population at high risk of osteoporotic fractures in the adult population.^33^ The observed associations were partly supportive of our initial hypothesis of a positive association between high birth weight, higher childhood BMI, and adolescent BMC/aBMD. Higher TB BMC and aBMD values in children (2 to 18 years) with overweight/obesity, compared with normal‐weight children, have been shown in other, mostly cross‐sectional studies.^15^ The importance of our study is its longitudinal design with data from birth up to 18 to 20 years of age. We observed that overweight/obesity at 2.5 years of age in girls and at 6.0 and 16.5 years of age in both boys and girls were associated with higher BMC/aBMD in late adolescence. This seems a positive finding because a 10% increase in peak aBMD is predicted to delay the development of osteoporosis in women by 13 years.^34^ However, it could be questioned whether the higher aBMD in individuals with overweight/obesity is sufficient given the excess weight load. Other studies have shown conflicting effects of obesity and excess fat on bone strength, and reported increased risk of fracture among overweight children.^16^, ^17^ We found indications that the effect of increasing BMI at 16.5 years of age on TB BMC and aBMD was leveling off when BMI exceeded 30 kg/m^2^ (Supplemental Figs. S1 and S2). However, there were few obese adolescents in our study population, limiting our ability to draw firm conclusions.

Both boys and girls with overweight/obesity at 16.5 years of age had significantly higher TH aBMD *Z*‐scores compared with those of normal weight. Greater weight gain in each period from age 2.5 years was also positively associated with higher TH aBMD. This supports the notion of adaption to mechanical loading.^15^, ^18^ Another study from this same cohort showed that tracking (stability) of overweight/obesity from childhood to 15 to 20 years of age was moderate to strong,^35^ so a high weight loading is likely to have persisted over time. Others have also found long‐term benefits of high childhood BMI on bone mass in adulthood, in addition to physical fitness.^36^ As reported by others, the effect of excess weight on bone might be site‐specific^37^, ^38^ and might in part be explained by increased lean mass.^18^, ^38^ In another cross‐sectional study of the TFF1 cohort,^39^ both fat mass and lean mass emerged as strong predictors of bone mass at femoral neck and total hip, with lean mass being the most influential. It also showed that in adolescents, especially girls with low lean mass, fat mass was more important.^39^ Clark and colleagues found positive associations between fat mass and bone mass and bone growth in prepubertal children and concluded that adipose tissue stimulates bone growth.^40^ Results from a Mendelian randomized study suggested that adiposity is causally related to increased aBMD in children, especially at weight‐bearing sites.^37^ Others have pointed to greater lean mass in overweight children, which may account for differences.^18^, ^41^ Because we do not have information on childhood body composition, we cannot distinguish between the potential different impacts of fat and lean mass in childhood. In this picture, it is, however, important to notice the consistent trend of being underweight during childhood and at 16.5 years of age was associated with lower BMC/aBMD *Z*‐scores at 15 to 20 years of age compared with those of normal weight, both in girls and boys. This is in line with findings from another study of Scandinavian children and adolescents.^42^ Previous studies have also shown an association between thinness in childhood and increased risk of hip fracture later in life.^12^, ^13^

Our results confirm earlier findings that a high rate of height and weight growth in early childhood is associated with higher bone mass at different ages later in life.^43^, ^44^ Gaining height faster than others between 6.0 and 16.5 years of age revealed lower effect estimates than faster growth in early childhood. This is in line with findings by Kuh and colleagues, who have studied bone measures in early old age.^44^ They found lower effect estimates for aBMD for height gain between 7 and 15 years than for height growth in early childhood. They found that hip aBMD was negatively associated with postpubertal height gain, especially in boys, explaining the findings with redistribution of bone as a biomechanical response to longitudinal growth.^44^ We found that length/height and weight gain at different age periods influenced bone measures at 15 to 20 years of age differently. How this may affect final achievement of peak bone mass and future fracture risk is not yet clear. Mikkola and colleagues who studied growth in individuals born between 1934 and 1944 found that in men, hip fracture risk in older age was driven by increase in height between 2 and 7 years of age and gain in BMI between 7 and 11 years of age. However, in women, early growth was not associated with the risk of hip fractures.^45^

In all analyses, higher effect estimates were found for BMC than for aBMD. This is not unexpected because height gain, especially during maturation, both influence BMC and bone size (bone area) and may as a consequence give lower aBMD if BMC does not increase proportionally more than bone area.^2^, ^4^, ^5^ This was most clearly observed for height growth between 6.0 to 16.5 years of age in boys, which resulted in significantly higher TH and TB BMC but negative aBMD. The limitations of the two‐dimensional DXA technique might also overestimate bone area in larger bones and hence underestimate aBMD.^2^

Overall, higher estimated BMC/aBMD *Z*‐scores were found for girls than for boys. However, no statistically significant sex difference was found when cross‐product terms with sex and the exposure variables were included in the models. Stronger associations with bone mass in girls have also been observed in several other studies.^10^, ^15^, ^41^ We know from other studies of the Fit Futures cohort that there are sex differences in the timing of skeletal growth and that especially the boys have probably not reached peak bone mass.^23^ Hormonal influence and pubertal timing may partly account for that, as suggested by others.^15^, ^41^ Pubertal maturation is likely a mediator in the relationship between weight in childhood and bone acquisition, as illustrated in another study.^41^

Birth weight was associated with higher TB BMC at 15 to 20 years of age in both sexes. The estimated effect was modest, 0.13 (95% CI 0.02 to 0.23) and 0.31 (95% CI 0.20 to 0.41) *Z*‐score higher TB BMC in boys and girls, respectively. This is in line with previous studies.^8^, ^10^, ^46^ This finding supports the importance of intrauterine skeletal development, as shown by others.^3^, ^8^, ^10^, ^43^ Associations between birth weight and bone strength parameters later in life have been contradictory.^9^, ^10^, ^11^ Youths in this cohort were born in a period with high mean birth weight in Norway,^20^ generally supporting sufficient nutrition in utero, which may limit our power to study maternal nutritional effects on later bone health. Information on maternal smoking and BMI was not available from the MBRN for this birth cohort, but we know from other MBRN data that few had any diseases. Generally, maternal health is good; undernutrition and severe malnutrition is rare among the Norwegian mothers of today.^20^, ^47^ Leunissen and colleagues found no influence of birth size on TB aBMD at 18 to 24 years of age and concluded that postnatal growth and weight gain were the main determinants.^11^ In our study, associations between birth weight and aBMD was only significant for TB in girls. Stronger associations were found with later height growth and weight gain, and adolescent bone mass accrual, than for birth weight and adolescent bone mass. After additional adjustment for BMI at 16.5 years of age, the effect of birth weight was no longer significant (data not shown). According to Lucas and colleagues,^48^ if the associations are attenuated or removed after adjustment for later size, later size is likely to be more relevant than early size. There is consistent evidence that both intrauterine life and childhood are important periods for foundations of later bone mass.^8^, ^9^, ^10^, ^11^, ^43^, ^44^ Although the majority of variance in peak bone mass is explained by genetic factors,^6^, ^7^ environmental factors at different ages are of importance.^4^ Sufficient maternal nutrition and healthy lifestyle during pregnancy and maintenance of a healthy weight through childhood all seem therefore important for the maximizing of peak bone mass. From other studies, we also know that physical activity plays an important role.^2^, ^36^ Even today, both undernutrition and malnutrition and obesity constitute a challenge related to optimal bone accrual. To promote bone health in adulthood, public health efforts should focus on these topics. This is recently highlighted by other researchers in the field of pediatric and adolescent bone health.^49^

The main strength of this study is its population‐based design and access to longitudinal data from birth to 18 to 20 years of age. The high attendance rate in TFF1 and the population‐based design reduce the risk of selection bias. Most studies of bone strength in children/adolescents are cross‐sectional studies; thus, longitudinal studies are called for.^15^, ^17^, ^18^ Our study is fairly large compared with others in the field.^10^, ^15^, ^17^, ^18^ This gave us the opportunity to stratify the analyses by sex to avoid biased estimates due to differences in bone growth between girls and boys, especially during adolescence.^3^, ^4^, ^18^ Data from the MBRN and objectively measured height/weight in childhood minimized the risk of information bias. Repeated DXA measurements were performed using the same instrument with a documented good precision^24^ to avoid systematic error in the outcome measures. Repeated measures of bone mass at 15 to 17 and 18 to 20 years of age are an advantage because BMC and aBMD increase during adolescence, which has been observed in this cohort.^23^

The main limitation in this study is the number of participants with missing data. Despite a high participation rate in TFF1 (>90%), this introduces a risk of selection bias. Because of the retrospective collection of exposure variables, missing data from birth and childhood are not dependent on the outcome. More boys than girls did not attend TFF2. However, dropout analyses did not indicate any other main differences between participants with and without missing data. Sensitivity analyses did not indicate that missing data were influential in our estimates. Linear spline multilevel modeling^25^ was used to predict length/height and weight at exact ages in childhood and estimate growth trajectories. This is a particularly useful method to deal with challenges when data are not measured at the same point in time, data are from different sources, and with missing values.^25^ A recognized method was used to impute missing covariates.^32^ We used linear mixed models, assumed to be robust against missing data,^50^ and missing data from TFF2 did not affect the number of participants included in the analyses because all available data were used. Another limitation is the lack of information on potential confounding factors, such as parental (genetic), nutritional, physical activity, and other lifestyle factors at birth and childhood that are known to affect skeletal development.^3^, ^4^ We cannot rule out the possibility of unmeasured confounders, making our models somewhat incomplete and open to residual confounding. Measures of BMC and aBMD with DXA are a proxy for bone strength,^2^ and DXA measurements have some limitations versus more sophisticated measures of bone strength like bone macro‐ and microarchitecture.^2^, ^5^ However, aBMD is estimated to predict 66% to 74% of the variation in bone strength^51^ and is the most frequently used measure in children and adolescents.^2^

In summary, we saw a positive association between high birth weight and BMC in adolescence. Length/height growth and weight gain in childhood revealed stronger associations with bone accrual at 15 to 20 years of age. We therefore conclude that birth weight has an effect on adolescent bone mass but less than later growth and BMI in childhood and at adolescence. Overall stronger associations were found for TB than for TH and stronger associations with BMC than with aBMD. Our findings did not indicate that overweight/obesity in childhood negatively affected bone mass accrual, but underweight was consistently associated with lower BMC and aBMD *Z*‐scores.

## Disclosures

All authors state that they have no conflicts of interest.

## Supporting information

Supporting Figure S1.

Supporting Figure S2.

Supporting Table S1.

Supporting Table S2.
