# Supplementary figures and images for: How Is Adolescent Bone Mass and Density Influenced by Early Life Body Size and Growth? The Tromsø Study: Fit Futures—A Longitudinal Cohort Study From Norway
Source: JBMR Plus. 2018 Jun 7;2(5):268–80. doi: 10.1002/jbm4.10049 (PMC6139726; doi:10.1002/jbm4.10049)

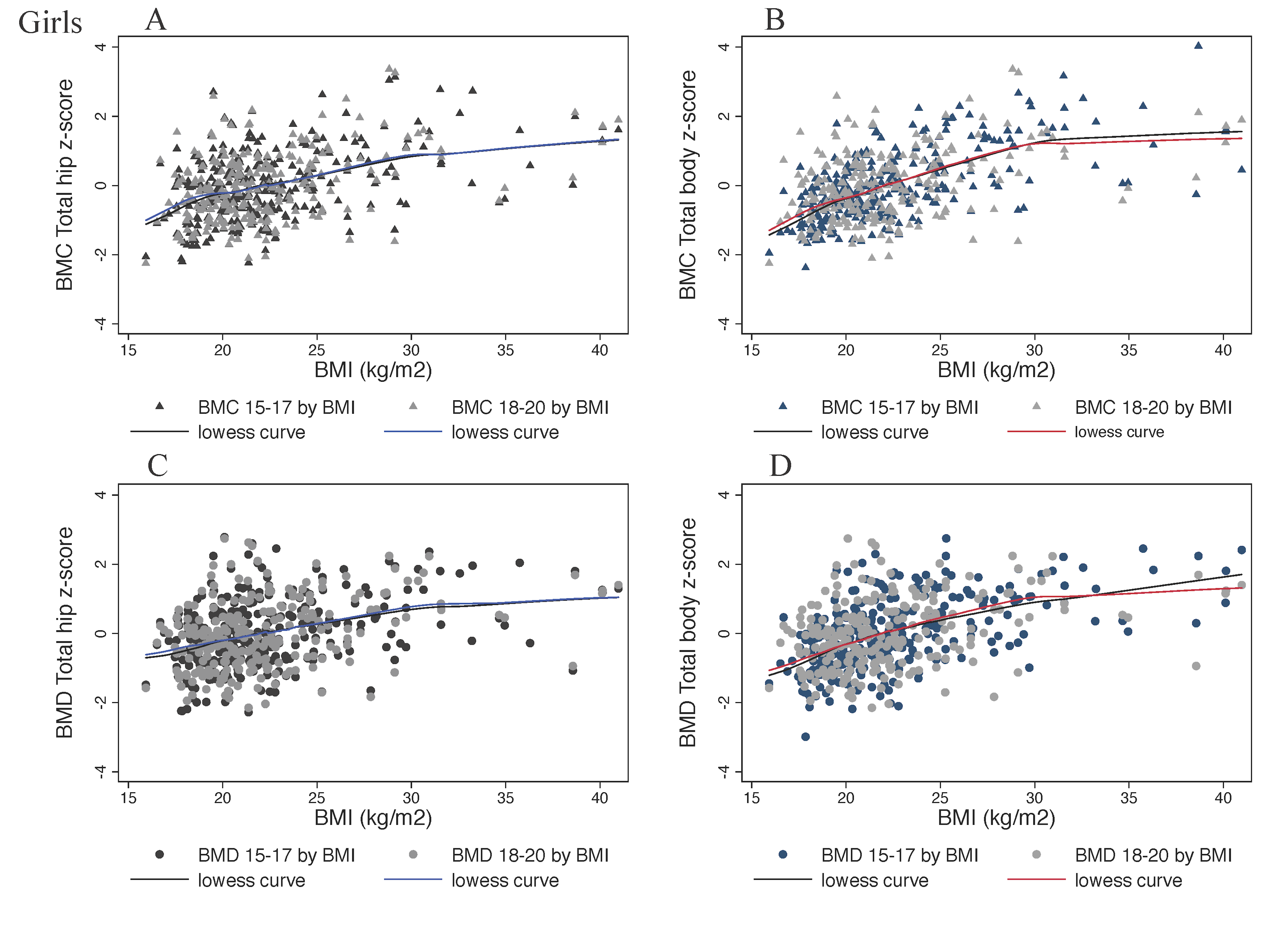

Supplement: Supplementary file 1 — Supporting Figure S1. [file JBM4-2-268-s001.tiff]

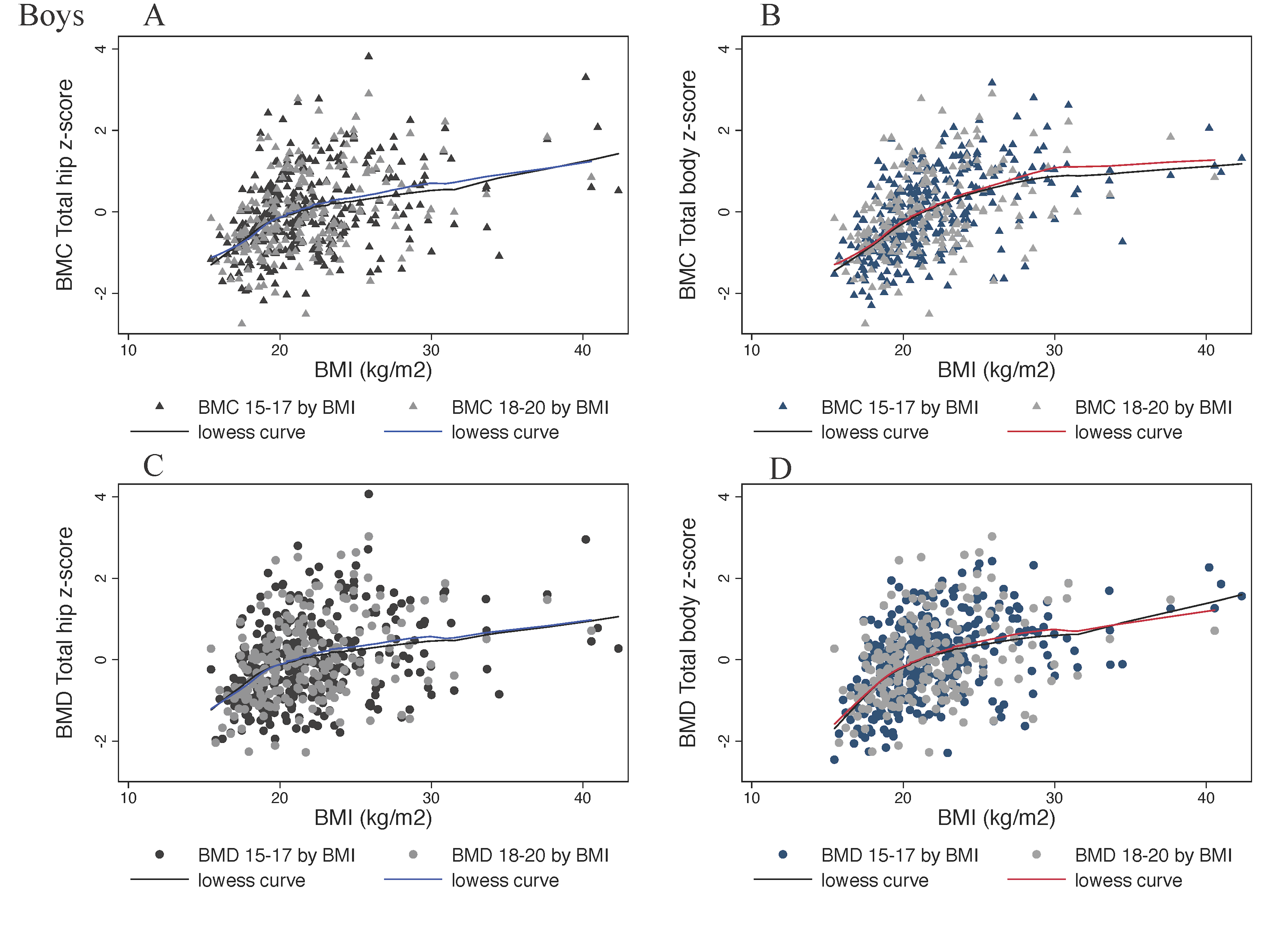

Supplement: Supplementary file 2 — Supporting Figure S2. [file JBM4-2-268-s002.tiff]
