# Supplementary material for: How Is Adolescent Bone Mass and Density Influenced by Early Life Body Size and Growth? The Tromsø Study: Fit Futures—A Longitudinal Cohort Study From Norway
Source: JBMR Plus. 2018 Jun 7;2(5):268–80. doi: 10.1002/jbm4.10049 (PMC6139726; doi:10.1002/jbm4.10049)
Supplement: Supplementary file 3 — Supporting Table S1. [file JBM4-2-268-s003.docx]

Supplemental table 1: Differences between observed measurements and those predicted by the linear spline multilevel model, for girls and boys in The Tromsø study: Fit Futures

|  | n | | Mean actual  measurement (SD) | | | | Mean predicted  measurement (SD) | | | | Mean difference (SD) | | | |
| --- | --- | --- | --- | --- | --- | --- | --- | --- | --- | --- | --- | --- | --- | --- |
| Age 2-4 years | 633 | | 2.6 | | (0.4) | | 2.5 | | - | | -0.1 | |  | |
| Age 5-7 years | 633 | | 6.0 | | (0.4) | | 6.0 | | - | | 0 | |  | |
| Age 15-17 years | 633 | | 16.6 | | (0.4) | | 16.5 | | - | | -0.1 | |  | |
|  |  | |  | |  | |  | |  | |  | |  | |
| Girls |  | |  | |  | |  | |  | |  | |  | |
| Weight models (kg) |  | |  | |  | |  | |  | |  | |  | |
| Birth weight | 306 | | 3.48 | | (0.58) | | 3.48 | | (0.55) | | -0.0002 | | (0.026) | |
| Weight 2-4 years | 306 | | 13.51 | | (1.69) | | 13.55 | | (1.55) | | 0.031 | | (1.09) | |
| Weight 5-7 years | 306 | | 21.87 | | (3.93) | | 21.58 | | (3.80) | | -0.30 ^**^ | | (1.38) | |
| Weight 15-17 years | 306 | | 61.57 | | (12.09) | | 61.91 | | (12.25) | | 0.34 ^***^ | | (1.16) | |
|  | | | | | | | | | | | | | | |
| Length/height models (cm) | | | | | | | | | | | | | | |
| Length at birth | 287 | | 49.44 | | (2.14) | | 49.47 | | (1.63) | | 0.023 | | (0.61) | |
| Length 2-4 years | 306 | | 91.27 | | (4.39) | | 91.63 | | (3.18) | | 0.36 | | (3.72) | |
| Length 5-7 years | 306 | | 116.75 | | (5.06) | | 117.08 | | (4.15) | | 0.32 ^*^ | | (2.39) | |
| Length 15-17 years | 306 | | 165.51 | | (6.09) | | 166.10 | | (6.02) | | 0.56 ^***^ | | (0.69) | |
|  | | | | | | | | | | | | | | |
| Body mass index (BMI kg/m^2^) | | | | | | | | | | | | | | |
| BMI at birth | 287 | | 14.30 | | (1.49) | | 14.30 | | (1.56) | | 0.005 | | (0.27) | |
| BMI 2-4 years | 306 | | 16.20 | | (1.39) | | 16.11 | | (1.37) | | -0.09 ^***^ | | (0.33) | |
| BMI 5-7 years | 306 | | 15.97 | | (2.10) | | 15.69 | | (2.20) | | -0.29 ^***^ | | (0.47) | |
| BMI 15-17 years | 306 | | 22.48 | | (4.29) | | 22.44 | | (4.32) | | -0.04 ^*^ | | (0.33) | |
|  | | | | | | | | | | | | | | |
| Boys |  |  | |  | |  | |  | |  | |  | |  |
|  | | | | | | | | | | | | | | |
| Weight models (kg) | | | | | | | | | | | | | | |
| Birth weight | 327 | | 3.57 | | (0.59) | | 3.58 | | (0.57) | | 0.0002 | | (0.027) | |
| Weight 2-4 years | 327 | | 14.11 | | (1.76) | | 14.19 | | (1.60) | | 0.074 | | (1.04) | |
| Weight 5-7 years | 327 | | 22.10 | | (3.55) | | 21.63 | | (3.30) | | -0.47 ^**^ | | (1.47) | |
| Weight 15-17 years | 327 | | 69.71 | | (14.42) | | 69.86 | | (14.57) | | 0.15 | | (1.45) | |
|  | | | | | | | | | | | | | | |
| Length/height models (cm) | | | | | | | | | | | | | | |
| Length at birth | 317 | | 50.03 | | (2.38) | | 50.05 | | (1.79) | | 0.021 | | (0.68) | |
| Length 2-4 years | 327 | | 92.77 | | (4.66) | | 93.14 | | (3.33) | | 0.36 | | (3.79) | |
| Length 5-7 years | 327 | | 117.99 | | (5.25) | | 118.00 | | (4.46) | | 0.01 | | (2.43) | |
| Length 15-17 years | 327 | | 177.14 | | (6.74) | | 177.74 | | (6.56) | | 0.60 ^***^ | | (1.16) | |
|  | | | | | | | | | | | | | | |
| Body mass index (BMI kg/m^2^) | | | | | | | | | | | | | | |
| BMI at birth | 317 | | 14.25 | | (1.52) | | 14.25 | | (1.61) | | -0.002 | | (0.29) | |
| BMI 2-4 years | 327 | | 16.36 | | (1.31) | | 16.32 | | (1.30) | | -0.04 ^*^ | | (0.33) | |
| BMI 5-7 years | 327 | | 15.81 | | (1.75) | | 15.48 | | (1.79) | | -0.33 ^***^ | | (0.48) | |
| BMI 15-17 years | 327 | | 22.17 | | (4.17) | | 22.07 | | (4.20) | | -0.1 ^***^ | | (0.33) | |
|  | | | | | | | | | | | | | | |
| Paired samples t-test; mean difference significantly different from zero  ^*^ p<0.05 ^**^ p<0.001 ^**^p<0.0001 | | | | | | | | | | | | | | |
