# Supplementary material for: How Is Adolescent Bone Mass and Density Influenced by Early Life Body Size and Growth? The Tromsø Study: Fit Futures—A Longitudinal Cohort Study From Norway
Source: JBMR Plus. 2018 Jun 7;2(5):268–80. doi: 10.1002/jbm4.10049 (PMC6139726; doi:10.1002/jbm4.10049)
Supplement: Supplementary file 4 — Supporting Table S2. [file JBM4-2-268-s004.docx]

| Characteristics | Girls | | | | | | Boys | | | | | |
| --- | --- | --- | --- | --- | --- | --- | --- | --- | --- | --- | --- | --- |
|  | Observed | | | Imputed ^a^ | | | Observed | | | Imputed ^a^ | | |
| *Birth* | n | Mean/% | (SD) | n | Mean/% | (SD) | n | Mean/% | (SD) | n | Mean/% | (SD) |
|  |  | |  |  | |  |  | |  |  | |  |
| Gestational age (weeks) | 272 | 39.8 | 1.8 | 306 | 39.8 | 1.7 | 303 | 39.5 | 2.1 | 327 | 39.5 | 2.1 |
| *15-17 years of age* |  |  |  |  |  |  |  |  |  |  |  |  |
| Menarche age | 302 | 13.0 | 1.2 | 306 | 13.0 | 1.2 | - | - | - | - | - | - |
| Pubertal maturation, girls ^b^ | 303 |  |  | 306 |  |  | - | - | - | - | - | - |
| Early (<12.5 years) | 85 | 28.1% |  |  | 28.2% |  | - | - | - | - | - | - |
| Intermediate (12.5-13.9 years) | 152 | 50.2% |  |  | 50.4% |  | - | - | - | - | - | - |
| Late (≥14.0 years) | 66 | 21.8% |  |  | 21.5% |  | - | - | - | - | - | - |
| Pubertal development scale (PDS) | - | - | - | - | - | - | 259 | 3.29 | 0.43 | 327 | 3.29 | 0.43 |
| Pubertal maturation, boys ^c^ | - | - | - | - | - | - | 259 |  |  | 327 |  |  |
| Barely started (PDS 2.0-2.9) | - | - | - | - | - | - | 46 | 17.8% |  |  | 17.8% |  |
| Underway (PDS 3.0-3.9) | - | - | - | - | - | - | 196 | 75.7% |  |  | 75.6% |  |
| Completed (PDS 4.0) | - | - | - | - | - | - | 17 | 6.6% |  |  | 6.6% |  |
| Physical activity – frequency | 303 |  |  | 306 |  |  | 321 |  |  | 327 |  |  |
| Low | 103 | 34.0% |  |  | 34.2% |  | 118 | 36.8% |  |  | 36.8% |  |
| Moderate | 133 | 43.9% |  |  | 43.8% |  | 117 | 36.5% |  |  | 36.4% |  |
| High | 67 | 22.1% |  |  | 22.0% |  | 86 | 26.8% |  |  | 26.8% |  |
|  |  |  |  |  |  |  |  |  |  |  |  |  |
| ^a^ Estimated values from a dataset with multiple (20) imputations, n= 306 girls and 327 boys.  ^b^ Pubertal maturation is based on age of menarche in girls.  ^c^ Pubertal maturation is based on PDS in boys.^(28)^ | | | | | | | | | | | | |

Supplemental table 2. Descriptive statistics for selected variables with missing data at birth and at 15-17 years of age, showing observed and imputed values for girls and boys, The Tromsø Study: Fit Futures
